# Psychophysiological effects of walking in forests and urban built environments with disparate road traffic noise exposure: study protocol of a randomized controlled trial

**DOI:** 10.1186/s40359-024-01720-x

**Published:** 2024-05-06

**Authors:** Julia Schaupp, Karin Hediger, Jean-Marc Wunderli, Beat Schäffer, Silvia Tobias, Natalia Kolecka, Nicole Bauer

**Affiliations:** 1 Swiss Federal Institute for Forest, Snow and Landscape Research (WSL), Birmensdorf, Switzerland; 2 Swiss Federal Laboratories for Materials Science and Technology, Dübendorf, Switzerland; 3 Faculty of Psychology, Department of Clinical Psychology and Psychotherapy, University of Basel, Basel, Switzerland; 4 Department of Epidemiology and Public Health, Swiss Tropical and Public Health Institute, Allschwil, Switzerland; 5 Faculty of Psychology, Open University, Heerlen, the Netherlands

**Keywords:** Greenspace, Forest, Urban built environment, Road traffic noise, Walking, Stress, Attention, Restoration, Wellbeing, Field experiment

## Abstract

**Background:**

Stress is a widespread phenomenon and reality of everyday life, entailing negative consequences for physical and psychological wellbeing. Previous studies have indicated that exposure to greenspaces and nature-based interventions are promising approaches to reducing stress and promoting restoration. However, an increasing percentage of the population lives in urban regions with limited opportunities to spend time in greenspaces. In addition, urban settings typically feature increased levels of noise, which represents a major environmental stressor. Although various studies have compared the effects of exposure to greenspaces versus urban built environments, evidence of the effects of noise in this context is very limited. Psychophysiological benefits of exposure to greenspaces compared to urban built environments reported in earlier studies might be less (or at least not only) due to features of the greenspaces than to additional stressors, such as road traffic noise in the urban built environment. Hence, differences in the effects attributed to greenness in previous studies may also be due to potentially detrimental noise effects in comparison settings. This paper reports the study protocol for a randomized, controlled intervention study comparing the effects of walking in forest versus urban built environments, taking road traffic noise exposure during walks in the respective settings into account.

**Methods:**

The protocol envisages a field study employing a pretest–posttest design to compare the effects of 30-min walks in urban built environments and forests with different road traffic noise levels. Assessments will consist of self-reported measures, physiological data (salivary cortisol and skin conductance), an attention test, and noise, as well as greenness measurements. The outcomes will be restoration, stress, positive and negative affect, attention, rumination, and nature connectedness.

**Discussion:**

The results will inform about the restorative effect of walking in general, of exposure to different types of environments, and to different noise levels in these sites. The study will provide insights into the benefits of walking and nature-based interventions, taking into account the potential detrimental effects of noise exposure. It will thus facilitate a better understanding of low-threshold interventions to prevent stress and foster wellbeing.

**Trial registration:**

ISRCTN48943261; Registered 23.11.2023.

**Supplementary Information:**

The online version contains supplementary material available at 10.1186/s40359-024-01720-x.

## Background and rationale

Stress is a widespread phenomenon and part of our everyday life, with negative consequences for physical and psychological wellbeing. Data from the Swiss Health Survey 2017 revealed that in 2017, 21% of the respondents stated suffering from stress at their workplace “always” or “most of the time,” which represents an increase of 3% since 2012 [1]. Respondents who stated feeling stressed very often had a higher risk for burnout and emotional fatigue, compared with respondents reporting less stress [2]. Stress increases the risk of cardiovascular disease [3–7], gastrointestinal disorders [8], and blood pressure elevation [9]. Furthermore, stress is a risk factor for diabetes [10], obesity [11], and dementia [12] and is associated with hypertension [13]. Thus, stress constitutes an important public health issue, and research concerning stress recovery and approaches to prevent mental distress and improve psychophysiological wellbeing are needed.

In response to this urgency, some studies indicate that exposure to greenspaces can reduce stress symptoms and improve restoration and wellbeing [14–19]. Thus, exposure to greenspaces and nature-based interventions may be promising approaches to reduce stress and promote restoration. However, an increasing percentage of the population lives in urban regions with limited opportunities to spend time in greenspaces. In addition, such urban settings typically feature increased levels of transportation noise, representing a major environmental stressor and global challenge [20, 21]. Accordingly, the maintenance of environments suitable for promoting wellbeing, such as greenspaces, is suggested to foster mental health and support possibilities for coping with stress [22–31].

Much of the recent research on the benefits of engaging with greenspaces has been guided by theories concerned with psychophysiological stress reduction (stress reduction theory) [32, 33] or directed attention restoration (attention restoration theory) [34, 35]. Attention restoration theory states that nature allows restoration from a depleted capacity to direct attention, thus enabling more effective cognitive performance [35]; stress reduction theory proposes that natural environments promote recovery from stress and decrease arousal and negative thoughts through psychophysiological pathways [32, 33]. Correspondingly, there is an interest in measuring psychophysiological and attentional responses to greenspaces.

Although various earlier studies have compared the restorative effects of exposure to greenspaces versus urban built environments [18, 36–39], evidence of the effects of noise in this context is very limited. In their meta-analysis, Bowler and colleagues [36] compared the added benefits to health and well-being outcomes from activities such as walking and running in greenspaces versus synthetic (outdoor and indoor-built) environments. The results were mixed and revealed a paucity of good-quality studies. The authors concluded that well-designed studies are needed to further strengthen evidence and noted that differences between the benefits of greenspaces and urban built environments are less due to features of the greenspaces than to additional stressors in the urban built environment–one such stressor possibly being traffic noise. At the same time, Van Renterghem [31] discusses how vegetation can mitigate environmental noise perception, proposing a complex interplay between vegetation and noise factors for restoration.

In this context, we argue that although various earlier studies have compared the effects of exposure to greenspaces versus urban built environments, not enough attention has been given to potential confounding variables in previous studies. The greenspaces and urban built environments in earlier studies may have differed not only in terms of the extent of greenness of the compared environments, but also in terms of other variables. One particular variable in which environments might have differed is the extent of transportation noise (in particular road traffic noise) in different settings. Hence, differences in the effects attributed to greenness in previous studies may also be attributed to potentially detrimental (road traffic) noise effects in comparison settings, which potentially confounded previous results.

One previous study aimed at comparable noise levels in different environments, namely that by Gidlow and colleagues [40], who compared the effects of 30-min walks on stress, cognitive performance, restoration, and mood in a greenspace with water, a greenspace without water, and a pleasant residential urban environment. Differences between the different environments were found only for some of the outcome variables. The study showed no differences in the decrease in cortisol and improvements in mood between the different environments. A higher restorative experience in greenspaces, compared to the residential urban environment, and better cognitive performance 30 min after walking in greenspaces, compared to the residential urban environment, was shown. However, noise was only measured at one representative point on each route in each environment (urban residential 50.6 ± 4.3 dBA, green 47.5 ± 2.9 dBA, blue 45.6 ± 1.5 dBA) with two 15-min recordings in this study but was not assessed continuously during the walks. Furthermore, a difference of 5 dB between the conditions may result in a noticeable difference in noise levels between the different environments.

Research on noise exposure has shown associations between environmental noise, noise annoyance, and stress [41–45]. Studies on the effects of long-term noise exposure report an association between long-term aircraft noise exposure and a modified cortisol circadian rhythm [46] and decreased annoyance and increased wellbeing after a reduction of road traffic noise [47]. In contrast, various other studies could not establish such effects. Stockholm and colleagues [48] investigated the effects of long-term occupational noise on off-work cortisol levels. Their study showed no significant association between noise and cortisol levels. Other studies by Michaud et al. [49, 50] examined the effect of wind turbine noise levels up to 46 dBA on stress and found no effect. Studies on the effects of short-term noise exposure evidenced increased salivary cortisol levels for participants asked to solve a cognitive task in a louder (90 dBA) environment compared to a less noise-exposed environment (55–60 dBA, [51]; 45 dBA, [52]). Ellermeier et al. [44] found that skin conductance increased with the sound pressure level of vehicle sounds in a laboratory experiment. Overall, there is some evidence for stress as an effect of noise, suggesting that noise may also function as an acute impediment for stress reduction and restoration, but many results are based on cross-sectional data [41, 43, 53, 54] or laboratory studies [44, 51, 55–57]. Field experiments have shown weaker effects than laboratory research and have often relied on a very small sample size (e.g., Barbaresco et al. [58]). Good-quality field studies concerning the acute effects of noise on stress are lacking.

The planned study addresses this research gap. It will contribute to the existing literature with field experiments in ecologically valid settings, investigating the acute effects of walking in greenspaces versus urban built environments with different road traffic noise exposures, employing a longitudinal design. Following the need for robust experimental examination of psychophysiological responses to greenspaces expressed by Bowler et al. [36], the study presented in this protocol examines the effects of exposure to greenspaces versus urban built environments, with minimum confounding effects associated with road traffic noise. In addition, it provides a factorial design to study the individual contributions of environment and road traffic noise to the psychophysiological responses. It thus compares the effects of walking in greenspaces versus urban built environments with high versus low traffic noise exposure.

The overarching aim of this study is to assess the restorative effect of exposure to different types of environments (forests and urban built) and to different road traffic noise levels in these sites (high and low traffic noise), thus providing an improved empirical foundation for: (1) the practical application of walking and nature-based interventions as low-threshold approaches for stress reduction and the promotion of wellbeing; (2) the effects of additional environmental qualities of greenspaces and urban built areas like noise as an impediment to recover from stress; and (3) urban planning and the construction and preservation of restorative environments that support the wellbeing of inhabitants. In this paper, the study protocol is presented, and we used the SPIRIT checklist when writing our report [59].

The planned study is embedded in the research project RESTORE (Restorative potential of green spaces in noise-polluted environments). The purpose of the RESTORE project is to study green spaces as facilitators for restoration and stress recovery, as well as road traffic noise as an impediment to recovering from stress. The study presented here is the basis of a dissertation project examining the acute psychophysiological effects of walking in different environments and with disparate inner attitudes. In addition to investigating how much individuals restore, depending on *where* they go for a walk (research focus 1, RF1; focus of this study protocol), the study also investigates the effects of *how* individuals go for a walk and which inner attitude they adopt while walking. Specifically, this dissertation project examines whether helping people be more mindful while walking in forests may increase the potential positive effects of walking in forests on restoration (research focus 2, RF2). The current study protocol, however, focuses on describing the planned experiment for RF1). First, by means of inspecting the effects of 30-min walks, we will examine whether walking in forest settings, compared to walking in urban built settings, leads to a stronger increase in stress reduction, restoration, positive affect, attention, nature connectedness, and rumination as well as a stronger decrease in negative affect. Second, we will assess whether walking in an environment with less road traffic noise, compared to walking in an environment with more road traffic noise, increases the above effects.

## Methods

### Study design

We will conduct a randomized, controlled intervention field study employing a pretest–posttest design to compare the effects of 30-min walks in urban built environments and forests with different road traffic noise levels. Healthy adults will be asked to go for a guided group walk with a maximum of six participants. The participants will be randomly assigned to one of the following conditions: urban built environment with high road traffic noise, urban built environment with low road traffic noise, forest with high road traffic noise, and forest with low road traffic noise. A fifth condition will be a walk in a forest with low traffic noise, in which participants walk with a mindfulness intervention. In this study protocol, we focus on the examination of the objective environmental factors, as noted above, and the last condition is not discussed here (above RF2). Data will be assessed before (t1) and directly after (t2) the walk. After this, participants will be instructed to walk individually three times during the subsequent 10 days. Ten days after participating in the field experiment, participants will receive an invitation to respond to a third questionnaire (t3). The third measurement point is only used for RF2 and is not included here; as for answering the research questions included in this study protocol, we focus only on measurement timepoints t1 and t2.

### Research questions and hypotheses

#### Research question RQ1

Compared to walking in an urban built environment, does walking in a forest lead to a stronger increase in stress reduction (variable 1), restoration (variable 2), positive affect (variable 3), attention (variable 4), nature connectedness (variable 5), and rumination (variable 6) as well as a stronger decrease in negative affect (variable 7)?

#### Hypothesis H1

Compared to walking in an urban built environment, taking a walk in a forest leads to a stronger increase in stress reduction (1), restoration (2), positive affect (3), attention (4), nature connectedness (5), and rumination (6) as well as a stronger decrease in negative affect (7).

#### RQ2

Compared to walking in an environment with high traffic noise, does walking in an environment with low traffic noise lead to a stronger increase in the outcome variables 1–6 as well as a stronger decrease in negative affect?

#### H2

Compared to walking in an environment with high road traffic noise, taking a walk in an environment with low road traffic noise leads to a stronger increase in the outcome variables 1–6 as well as a stronger decrease in negative affect.

#### RQ3

Compared to walking in a forest with high road traffic noise, does walking in a forest with low road traffic noise lead to a stronger increase in the outcome variables 1–6 as well as a stronger decrease in negative affect?

#### H3

Compared to walking in a forest with high road traffic noise, taking a walk in a forest with low road traffic noise leads to a stronger increase in the outcome variables 1–6 as well as a stronger decrease in negative affect.

#### RQ4

Compared to walking in an urban built environment with high road traffic noise, does walking in an urban built environment with low road traffic noise lead to a stronger increase in the outcome variables 1–6 as well as a decrease in negative affect?

#### H4

Compared to walking in an urban built environment with high road traffic noise, taking a walk in an urban built environment with low road traffic noise leads to a stronger increase in the outcome variables 1–6 as well as a stronger decrease in negative affect.

#### RQ5

What is the effect of 30-min walks in the forest on outcome variables 1–6 compared to walking in urban built settings, depending on the noise in the settings?

### Procedures

The field experiment is based on a between-subject design, and assessments consist of self-report questionnaires, physiological measures, an attention test, and objective noise measurements. In preparation for the main data collection, a pretest was conducted in February and March 2022 to test the questionnaire and determine if changes in the experimental procedure were required. The questionnaire and experimental procedure were adjusted in line with the outcomes of the pretest.

Walking sessions will take place between 2:00 p.m. and 5:30 p.m., since participants’ salivary cortisol levels will be assessed, and salivary cortisol levels have been shown to fluctuate systematically during the day [60–62]. Additionally, stronger mental fatigue during the daytime is assumed [63] and hence, a higher need for restoration [64].

Before participation, individuals will be given a short introduction to the research topic, their task in the experiment, privacy policies, and asked to give their written informed consent. Before the date of participation, individuals will be asked to refrain from consuming caffeine and from performing exhausting physical activity 2 h before their walking appointment. Furthermore, participants will be requested to refrain from going for a walk on the day of their walking appointment before their participation and not to consume any drugs 24 h before taking the walk.

**Fig. 1:**
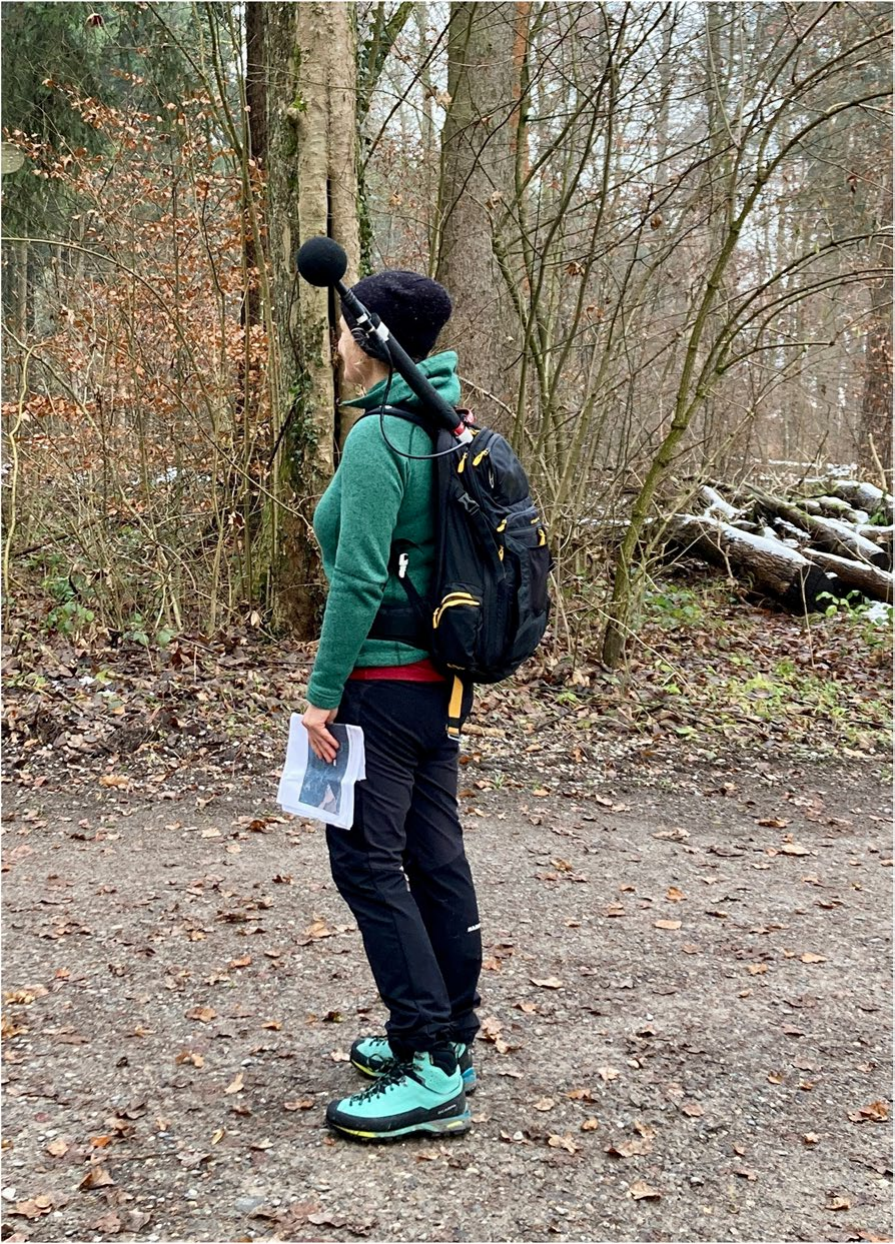
Audio recording

Upon arrival at the test site, the participants’ salivary cortisol levels will be assessed to obtain a baseline stress level. Next, participants will complete a series of questionnaires. After this, participants will be equipped with wearable wristbands to assess their skin conductance during the walk. Then, the participants’ attention will be assessed using the Necker Cube Pattern Control Test [65, 66]. Before starting the walk, a 2-min baseline value of participants’ skin conductance will be obtained while the individuals are seated, and a second salivary cortisol probe will be taken directly before the walk starts. One of the two experimenters present in the test setting will then lead the participants at a moderate pace of around 4,7 km/h along a predetermined route. The second experimenter will stay at the starting point of the route and look out for the assessment devices. Skin conductance will be measured continuously during the walk. Furthermore, during every walk, the experimenter will measure the sound while walking about 6 m ahead of the participants. A sound level meter (XL2 by NTI Audio, Schaan, Liechtenstein) with a free-field measurement microphone, fulfilling class 1 environmental requirements according to IEC 61672, will be used. The microphone will be fixated onto a recording stick and carried in a backpack during the walk by the experimenter, resulting in a microphone height of approximately 1.7 m above the ground (see Fig. 1).

**Fig. 2:**
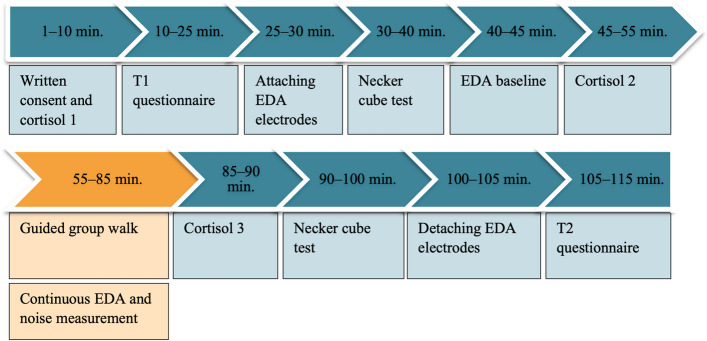
Study procedure

Participants will be asked to walk in silence and not to outpace the experimenter. After the walk, a third cortisol probe will be taken, the attention test will be conducted again, and participants will be asked to respond to the post-questionnaire. For an illustration of the study procedure, see Fig. 2.

Data will be collected in three waves: May to October 2022, August to October 2023, and in a third wave in 2024. Any substantial protocol modifications will be communicated to *BMC Psychology* and approved by the ethics committee.

### Outcomes

#### Primary outcomes

The primary outcomes are “perceived restoration”, measured with a self-report questionnaire and salivary cortisol as a physiological stress marker.

Perceived restoration will be assessed using a slightly modified version of the restoration outcome scale (ROS) [67]. The questionnaire was put in past tense, and participants will be asked to what extent the walk contributed to three items concerning relaxation and calmness (e.g., “I feel calmer after being here”), one item reflecting attention restoration (“my concentration and alertness clearly increased”), and two items indicating clearing one’s thoughts (e.g., “I was able to forget my everyday worries here”). The questionnaire has been translated into German, including a back translation into English by the internal translator of the Swiss Federal Research Institute WSL. Items are rated on a Likert scale ranging from 1 (*not at all*) to 7 (*completely*). The scale has been used in several previous studies examining the benefits of exposure to nature [14, 68, 69] and has been shown to have good internal consistency with a Cronbach’s alpha of .92 [67].

The physiological stress marker salivary cortisol is considered a reliable indicator for assessing hypothalamic pituitary adrenocortical (HPA) axis activity [62, 70]. Saliva samples will be collected using synthetic swabs (Sarstedt, Germany). For saliva collection, participants will place the swab in their mouth for 2 min before returning it to the salivette collection tube. The salivettes will be put in a portable cooler immediately after saliva collection and stored there until the end of the walk. When returning from the field, saliva samples are frozen and stored at − 20 °C until analysis. The samples will be analyzed by the laboratory of Professor Dr. Kirschbaum at the Technical University of Dresden, Germany, to determine cortisol levels. After thawing, the samples will be centrifuged at 3,000 rpm for 5 min to obtain a clear supernatant of low viscosity. Salivary concentrations are measured using a commercially available chemiluminescence immunoassay with high sensitivity (Tecan – IBL International, Hamburg, Germany; catalog number R62111).

#### Secondary outcomes

Self-report questionnaires

*Affect*

Positive and negative affect will be assessed using the Positive and Negative Affect Schedule [71]. It consists of 20 items, providing separate scores for a positive affect scale (10 items, e.g., “active” and “enthusiastic”) and a negative affect scale (10 items, e.g., “nervous” and “distressed”). Items are rated on a 5-point Likert scale ranging from 1 (*not at all*) to 5 (*extremely*). Participants will be asked to indicate the extent to which they currently experience each of the feelings or emotions. The scale has been widely used in previous studies examining the benefits of exposure to nature and has been shown to be sensitive to change in this context [14, 15, 72–74]. A good internal consistency of the scale, with a Cronbach’s alpha of between .86 and .90 for positive affect and between .84 and .87 for negative affect, has been reported [71].

*Rumination*

Rumination will be assessed using the German version of the Perseverative Thinking Questionnaire (PTQ; [75]). The PTQ measures content-independent repetitive negative thinking (RNT), which is referred to as rumination in research [76]. For 15 statements related to their process of thinking, the participants will rate how often they apply to them using a 5-point Likert scale ranging from 0 (*never*) to 4 (*almost always*). Item examples of RNT are: “The same thoughts keep going through my mind again and again”, or “Thoughts come to my mind without me wanting them to.” The internal consistency and convergent validity of the PTQ have been shown to be good [75]. The PTQ was evaluated in nonclinical and clinical samples and shown to have a high internal consistency of Cronbach’s alpha = .94 for the nonclinical sample [75].

*Nature relatedness*

Nature relatedness will be measured with the 6-item short form of the Nature Relatedness Scale (NR-6; [77]). The construct of nature relatedness [76] is assumed to capture an individual’s interest in nature, fascination with nature, and desired contact with nature. Nisbet and Zelenski [77] understand nature relatedness as encompassing an awareness and understanding of all aspects of the natural world, transcending aesthetically appealing aspects. In the present study, we will apply the German version [78] of the NR-6 (e.g., “I always think about how my actions affect the environment,” “My relationship to nature is an important part of who I am,” and “My ideal vacation spot would be a remote, wilderness area”). The scale encompasses the affective and cognitive elements of the relationship with nature. Participants will be asked to respond to the items on a 5-point Likert scale ranging from 1 (*strongly disagree*) to 5 (*strongly agree*). The NR-6 scale has been shown to have good internal consistency, with a Cronbach’s alpha between .83 and .86 [77].

*Connection to nature*

Connection to nature will be assessed using the Love and Care for Nature Scale (LCS; [79]). The LCS measures the emotional components of the connection between humans and nature with reference to the two terms love and care for nature. The initial version of the LCS was developed by Perkins [79]. Perkins defines the construct of love and care for nature as “deep love and caring for nature which includes a clear recognition of nature’s intrinsic value as well as a personal sense of responsibility to protect it from harm” [79]. The LCS was chosen due to its affective focus on the relationship with nature. In the present study, we will use the 5-item version of the scale. Items (e.g., “I feel a deep love for nature”) are rated on a 7-point Likert scale ranging from 1 (*strongly disagree*) to 7 (*strongly agree*). The scale has been shown to have good internal consistency, with a Cronbach’s alpha of .93 [79].

*Satisfaction with life*

Life satisfaction will be measured with the German General Life Satisfaction Short Scale (L-1; [80]); for the English version see Nießen an colleagues [81]). This constitutes a single item (“How satisfied are you at present, all in all, with your life?”), rated on an 11-point scale (0 = *not satisfied at all* to 10 = *completely satisfied*). A good test–retest reliability as well as high convergent validity with other multi-item scales measuring satisfaction with life have been shown for the L-1 [80].

*Perceived restorative qualities of environments*

The extent to which the different environments examined in this study have restorative qualities will be assessed using the Perceived Restorativeness Scale-11 (PRS-11) [82]. Pasini et al. [82] established an 11-item version of the scale, favoring a four-factor model with the factors “being away,” “fascination,” “coherence,” and “scope.” Results have shown the PRS-11 to discriminate among different environmental categories, with hills and lakes exhibiting the highest PRS scores and industrial zones holding the lowest scores [82]. The questionnaire has been translated into German, including a back translation into English by the internal translator of the Swiss Federal Research Institute WSL. Subjects rate the extent to which the items reflect their experience of the environment in which they have been walking with higher sum scores, indicating stronger perceived restorativeness. Information on the internal consistency for this scale is not available.

*Stress recovery*

In an additional question, using a 7-point Likert scale, ranging from 1 (*not at all*) to 7 (*completely*), participants indicated to what extent the environment they walked through contributed to them being able to recover from stress.

*Noise annoyance*

Noise annoyance will be assessed with the 11-point International Commission on Biological Effects of Noise (ICBEN) [83], also recommended in Standard ISO/TS 15666 [84], assessing per noise source how much the participants felt bothered, disturbed, or annoyed by noise from the corresponding sources during the walk (road traffic noise, public transport, railway noise, aircraft noise, construction work noise, or noise from restaurants, bars, or leisure activities). Items are rated on a Likert scale ranging from 0 (*not at all*) to 10 (*extremely*). The scale is a standardized noise annoyance scale widely used in noise annoyance research, yielding internationally comparable measures of annoyance reactions in noise studies [83].

*Acoustic impression of the soundscapes*

The participants’ individual experience of soundscapes will be assessed in accordance with the International Organization for Standardization data collection and reporting requirements ISO/TS 12913–2 [85]. Participants will rate the extent to which the soundscape during the walk was perceived as pleasant, chaotic, vibrant, uneventful, calm, annoying, eventful, and monotonous on a 5-point Likert scale ranging from 1 (*not at all*) to 5 (*completely*). Additionally, we added “loud” as a ninth attribute to rate on the same scale. Following ISO/TS 12913–2 [85], participants will further be asked to rate to what extent they heard the four following types of sounds during the walk: “traffic noise (e.g., cars, buses, trains, and airplanes),” “other noise (e.g., sirens, construction, industry, and loading of goods),” “sounds from human beings (e.g., conversation, laughter, children at play, and footsteps),” and “natural sounds (e.g., singing birds, flowing water, and wind in vegetation)”. Items will be rated on a 5-item Likert scale, ranging from 1 (*not at all*) to 5 (*dominates completely*). In addition, participants will be asked to rate the overall sound environment during the walk on a 10-point Likert scale, ranging from *very pleasant* to *very unpleasant*.

*Demographics*

We will assess age, gender, highest education, main occupation, income, frequency of exposure to nature, previous mindfulness experience, and noise sensitivity. Noise sensitivity will be evaluated with one item, asking participants how strongly they agree with the statement “I am noise sensitive”. The item is rated on a 5-point rating scale ranging from 1 (*do not agree at all*) to 5 (*very much agree*). The approach to assess noise sensitivity with a single question was taken from Brink et al. [86], who introduced this concept. Furthermore, we will ask the participants to describe their emotions through the sounds they heard during the walk. Finally, they will be asked to what extent going for a walk helps them think through personal problems and how their thoughts change when they go for a walk.

Physiological outcome measure

*Skin conductance*

Skin conductance level (SCL) will be measured continuously throughout the experiment with Shimmer3 GSR + sensor wristbands [87]. Electrodes will be placed on the volar phalanges of two fingers of the non-dominant hand. In previous research, SCL was shown to be an effective indicator of physiological stress, and SCL variations reflect changes in arousal [88].

Cognitive test

*Attention*

Attention will be assessed with the Necker Cube Pattern Control Test [65, 66], subsequently referred to as the Necker Cube Test. Stevenson et al. [89] and Ohly et al. [90] identified cognitive domains sensitive to restorative processes through exposure to greenspaces. According to Stevenson et al. [89] measures of attention control might exhibit the most direct relation to effortful directed attention. Here, different tests assessing attention control were explored, and the Necker Cube Test was selected [65]. The Necker Cube Test is a three-dimensional wire frame drawing of a cube without depth cues, which can be seen from two different perspectives [65]. When observed for more than a few seconds, the cube spontaneously switches perspectives due to reversals of the fore- and background [65]. It is assumed that holding one perspective and avoiding reversals requires directed attentional capacities [65]. After having been familiarized with the functionality of the paper version of the test, the participants are instructed to hold one perspective as long as they can and indicate every time the pattern reverses. Changes in perspective occurring despite attempting to focus on one pattern are believed to rely on attentional fatigue [35]. The number of reversals is thus used as a measure for directed attention, with fewer shifts indicating better attention.

### Test site selection and characterization

#### Selection of the test sites

First, we aimed to identify areas featuring a high variation in traffic noise at different times of the day to compare the benefits of walking in the same area with high and low traffic noise exposure. For this purpose, we analyzed traffic data from the city of Zurich, Switzerland, on the number of motorized individual vehicles as a proxy for traffic noise in the corresponding area [91]. The city of Zurich provides daily updated data on motorized individual vehicles in Zurich, measured at 97 measurement points of the traffic department (for the exact locations of the measurement points, see City of Zurich traffic department [92]). We randomly chose the working days of one week in 2021 (05.31.2021–06.04.2021) and calculated the mean number of vehicles per hour of the working days of this week from 8:00 a.m. to 8:00 p.m. We then checked the variances to identify the places with the highest hourly variation. Using this approach, we did not find settings with a stable time and a sufficiently high traffic noise variation of at least 10 dB between 8:00 a.m and 8:00 p.m. Thus, we decided to instead compare urban built and forest settings with widely constant noise conditions during the afternoon, featuring either high or low road traffic noise, which are as similar as possible between locations with respect to other characteristics besides noise (e.g., amount of vegetation, density of buildings, inclination, and presence of water).

To minimize the risk of systematically confounding loud and quiet settings with other variables that might potentially vary between settings with different noise levels (e.g., inclination in the walking route and flowing water in the surroundings), we selected three settings per condition in Zurich (three loud and three quiet urban built settings as well as three loud and three quiet forest settings). The test settings were selected stepwise. We incorporated geographic information system (GIS) data and GIS methods to select potential test sites and considered two important factors: road traffic noise and vegetation.

The road traffic noise data were obtained from the Swiss-wide noise database sonBASE for the year 2015 [93], and we used the mean exposure values for the daytime period between 6:00 a.m. and 10:00 p.m. SonBASE provides so-called rating sound levels (*Lr*) according to the Swiss Noise Abatement Ordiance (NAO) [94], with a spatial resolution of 10 × 10 m. The *Lr* corresponds to the A-weighted equivalent continuous sound pressure level (*L*_Aeq_) on a yearly average for road traffic, railway and aircraft noise, potentially with level corrections depending on the noise source and traffic density (for details see NAO [94]). To choose potential settings with similar noise characteristics, road traffic noise was divided into three noise classes (N1: Lr of daytime < 35 dBA, N2: Lr of daytime at 35–45 dBA, N3: Lr of daytime > 45 dBA). Areas with railway and aircraft noise *L*_r_ > 30 dB were excluded. Vegetation was characterized using its height (vegetation height model, VHM, [95]) as well as its abundance expressed by the normalized difference vegetation index (NDVI) [96], with NDVI values ranging from − 1 to 1 (NDVI > 0 shows vegetation), and VHM values given in meters. We calculated the median and standard deviation of the NDVI from all available cloud-free Sentinel-2 satellite images available between April and October for the years 2016–2019. Three classes of vegetation types were distinguished. To build the classes, we aimed to distinguish areas of similar characteristics in terms of NDVI and VHM based on unsupervised classification, using the Iso Cluster [97] and Maximum Likelihood Classification tools [98]. The classes were as follows: G1: hardly green spaces (mostly artificial surfaces); NDVI median = 0.07–0.24, VHM median = 0 m; G2: green spaces with low vegetation (mostly grass, with possible single shrubs and very small trees); NDVI median = 0.55–0.66, VHM median = 0.25–0.43 m; G3: green spaces with abundant and high vegetation (composed of trees, shrubs, and grass); NDVI median = 0.67–0.70, VHM median = 17.71–23.06 m.

The combinations of the three noise-based and the three vegetation-based categories indicated the potential test settings to be explored. In the next step, potential test sites were explored further via satellite images, inspecting the size of the areas (large enough for 30-min walks), vegetation, the existence of continuous sidewalks in the urban built areas, density, and height of buildings. Areas with water bodies or bridges were excluded, and we avoided steep road or path sections to minimize differences in exercise intensity. Finally, the remaining settings were inspected on-site. To avoid differences in social interaction between the settings, places with construction work were excluded as were places likely to feature large numbers of people. Potential walking routes were developed for suitable settings, and the sound pressure levels during the entire 30-min routes of the potential test settings were recorded during various walks at different times of the afternoon to inspect if the settings differed sufficiently in terms of noise. We aimed at a difference of 10 dB between loud and quiet settings. All of these criteria were used only for identifying potential and selecting the final test sites. In the final analysis, the actual measured noise levels during the walks will be taken as inputs for the statistical analysis.

#### Characterization of the final test sites

**Fig. 3:**
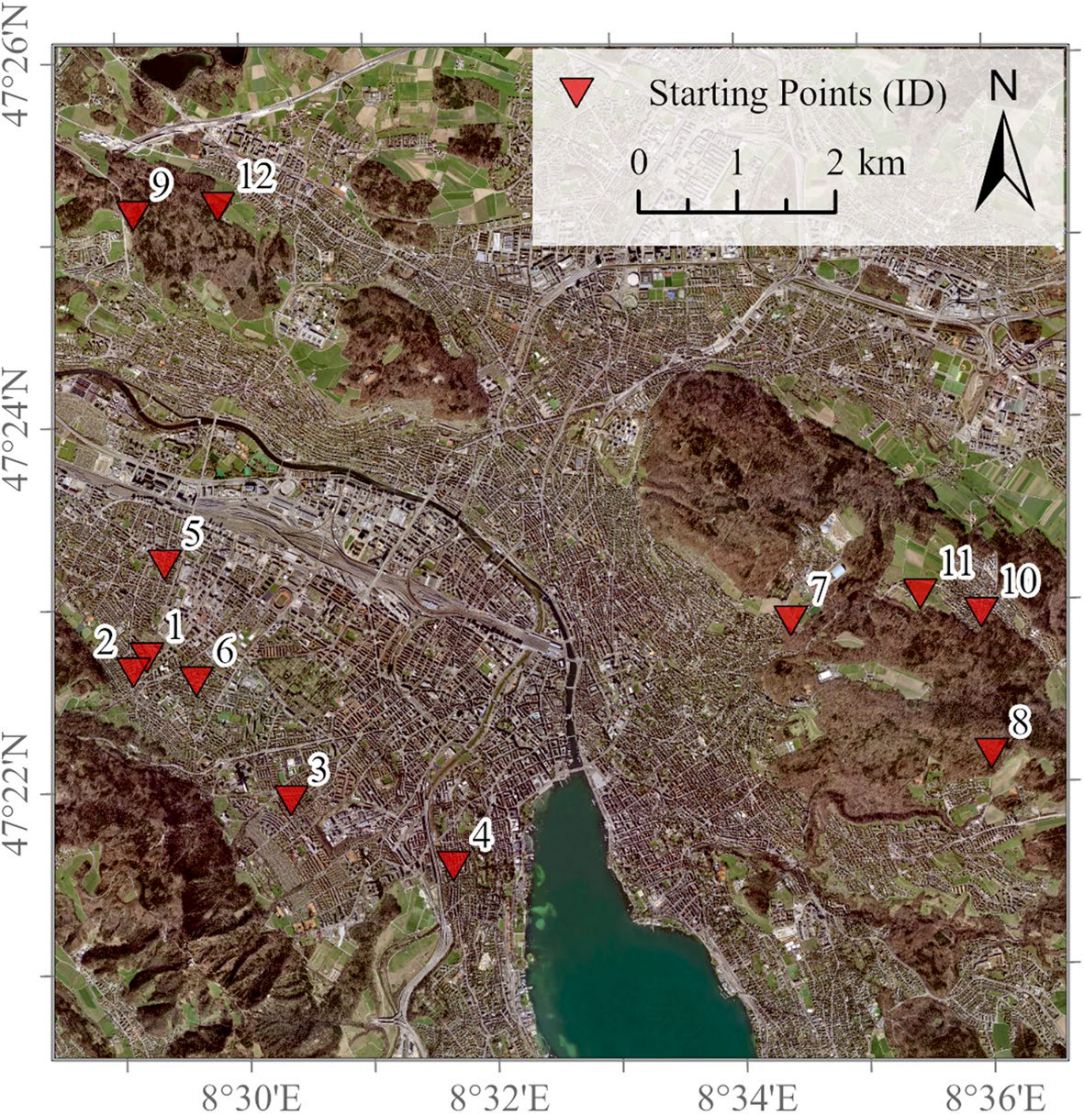
Locations of the final test settings. 1) Wydlerweg, 8047 Zurich. 2) Albisriederstr., 8047 Zurich. 3) Schweighofstr. 8055 Zurich. 4) Rieterplatz, 8002 Zurich. 5) Saumackerstr., 8048 Zurich. 6) Langgrütstr. 137, 8047 Zurich. 7) Krähbühlweg, 8044 Zurich. 8) Bruderholzweg, 8053 Zurich. 9) Alte Regensdorferstr., 8049 Zurich. 10) Hermann-Trüb-Weg, 8044 Dübendorf. 11) Forsthausweg, 8044 Zurich. 12) Hungerbergstr., 8049 Zurich. Basemap: swisstopo. 2020; Orthophoto (SWISSIMAGE 10) [99]

Figure 3 shows the locations of the starting points of the walking routes in the final test sites in the city of Zurich. For the exact coordinates of the starting points, see the file on “Locations of the starting points of the walking routes in the test settings” in the Supporting Information. The GPS files of the walking routes can also be found in the supporting information.

After selecting the final test settings, we performed a post-hoc classification of their vegetation and sound characteristics in the final test settings. For predicting vegetation, we calculated NDVI and VHM along each walking route with 50-m wide buffer zones on both sides of the lines. Table 1 shows a visual impression of the test settings and gives information on the vegetation characteristics of the respective walking routes. The locations for the two pictures on each route were chosen to be as characteristic as possible in terms of the visual and acoustical impressions of the walk.

**Table 1:** Characterization of vegetation in the final test sites

**Setting number**	**Picture place 1 in setting**	**Picture place 2 in setting**	**VHM**^**a**^** in meter on walking route with 50 m buffer**		**NDVI**^**b**^** on walking route with 50 m buffer**	
			***M***	***SD***	***M***	***SD***
1: UBHT^c^			1.42	3.33	0.35	0.13
2: UBHT			1.47	3.19	0.38	0.12
3: UBHT			1.63	3.27	0.37	0.14
4: UBLT^d^			2.18	4.49	0.28	0.13
5: UBLT			1.58	3.48	0.37	0.12
6: UBLT			2.14	4.25	0.37	0.14
7: FHT^e^			20.65	9.99	0.68	0.07
8: FHT			18.36	11.05	0.69	0.06
9: FHT			16.17	10.20	0.66	0.09
10: FLT^f^			24.27	8.47	0.70	0.05
11: FLT			22.19	9.90	0.69	0.05
12: FLT			20.00	8.41	0.7	0.04

Notes. ^a^VHM values in meters, calculated along each walking path, with 50-m wide buffer zones on both sides of the lines. Mean and standard deviation for the average maximum height of vegetation in the buffer zone. ^b^Median and SD NDVI values, calculated along each walking path, with 50-m wide buffer zones on both sides of the lines. ^c^UBHT= urban built with high traffic noise. ^d^UBLT= urban built with low traffic noise. ^e^FHT = forest with high traffic noise. ^f^FLT = forest with low traffic noise

Table 2 provides the acoustical and soundscape characteristics of the final settings. The first column shows the *L*_Aeq_ recorded on the walking routes before starting with the data collection. For an additional post hoc classification of the sound environment in the settings, the acoustic impression was evaluated for the two locations at each test site in the afternoons of the same week in 2023 by a civil servant not familiar with the research questions of the present study. The sound environment was evaluated three subsequent times for the last 5 min at every location. When analyzing the results of the sound evaluation, the mean and standard deviation of all six sound evaluations (2 locations × 3 replicate evaluations) in each setting were computed. Table 2 shows the results of the major items for evaluating the sound environment. An additional result of the post hoc classification of the sound environment was that, in urban built and forest settings with high road traffic noise, road traffic noise was judged the most dominant sound source in all settings. In urban built settings with low road traffic noise, road traffic noise or sounds from humans were judged as the most dominant sound source. In forest settings with little road traffic noise, natural sounds and sounds from other than the three previously mentioned sound sources were judged as most dominant. As intended, urban built settings with high road traffic noise showed a higher *L*_Aeq_ and were judged to feature a higher extent of traffic noise than urban built settings with low road traffic noise. Equally, forest settings with high road traffic noise showed a higher *L*_Aeq_ and were judged to feature a higher extent of traffic noise than forest settings with low road traffic noise. Generally, *L*_Aeq_ was higher in urban built settings, compared to forest settings and the extent of traffic noise was similar in urban built and forest settings. For urban built sites with (very) little vegetation and low noise, we could not find suitable environments with the dBA thresholds that we chose at the beginning. Thus, the thresholds had to be adapted to the environments present in the area of Zurich. This is because we strived for high external validity. Loud and quiet are thus defined depending on the environment. As expected, the extent of natural sounds was generally higher in forest settings, compared to urban built settings.

**Table 2:** Sound characterization of the final test sites

**Setting number**	***L***_**Aeq**_** on walking route**^a^		**Extent of traffic noise**^c^		**Extent of natural sounds**^d^	
	***M***	***SD***	***M***	***SD***	***M***	***SD***
1: UBHT^e^	68.71 (*n* = 3)^b^	3.55	3.50	0.55	1.83	0.41
2: UBHT	64.19 (*n* = 4)	1.67	2.50	0.84	1.83	0.41
3: UBHT	65.53 (*n* = 5)	1.33	3.00	0.00	1.00	0.00
4: UBLT^f^	58.42 (*n* = 4)	3.46	2.17	0.41	2.17	0.41
5: UBLT	57.53 (*n* = 5)	1.16	2.00	0.00	2.00	0.00
6: UBLT	48.74 (*n* = 2)	6.70	1.83	0.41	2.67	0.52
7: FHT^g^	64.30 (*n* = 6)	1.83	3.17	0.41	2.17	0.41
8: FHT	59.25 (*n* = 3)	1.11	3.67	0.52	2.33	0.52
9: FHT	59.55 (*n* = 4)	.87	3.50	0.55	2.17	0.41
10: FLT^h^	49.67 (*n* = 3)	1.80	1.83	0.75	3.33	0.52
11: FLT	49.98 (*n* = 3)	.61	1.17	0.41	3.50	0.55
12: FLT	49.98 (*n* = 4)	2.83	2.17	0.75	3.83	0.41

^a^L_Aeq_ of sound recording, measured on each route. ^b^Number of sound recordings in the setting. ^c^Extent to which traffic noise was heard during the last 5 min, responding on a Likert scale ranging from 1 (*not at all*) to 5 (*completely dominating*), evaluated by civil servant. ^d^Extent to which natural sounds were heard during the last 5 min, responding on a Likert scale ranging from 1 (*not at all*) to 5 (*completely dominating*), evaluated by a civil servant. ^e^UBHT urban built with high traffic noise. ^f^UBLT urban built with low traffic noise. ^g^FHT forest with high traffic noise. ^h^FLT forest with low traffic noise

For including noise as a predictor in the final statistical model, we will calculate the percentage of time in which traffic noise is the dominating sound source during each walk as well as the *L*_Aeq_ during the time in which traffic noise is the dominating sound source after having completed data collection.

### Participants and recruitment

Participants will be recruited by various means. A random sample of 7,000 people will be drawn from the residents’ registration office in the city of Zurich. The sample will be stratified over four age groups, 18–35, 35–50, 50–65, and > 65, to reach people of all ages. These participants will be invited to take part in the study by letter. In addition, students from the universities of Zurich (University of Zurich and Swiss Federal Institute of Technology Zurich) will be recruited via e-mail and by lecturers, who will forward the invitation to their students. The invitation to participate will also be distributed via local newspapers, local web portals, as well as at some of the authors' institution Empa. Furthermore, participants will be recruited through advertising with invitation flyers in public places in Zurich and via a study webpage. Finally, participants will be invited via the snowball sampling method, in which the researchers reach out to their social networks to distribute the invitation in Zurich. Participants will receive a remuneration of 50 CHF. Additionally, two massage vouchers amounting to 120 CHF each will be raffled anonymously among all participants who have completed the t1, t2, and t3 questionnaires. Individuals will be eligible for participation if they: (a) are 18 years and older; (b) are physically able to walk for half an hour at a moderate pace; (c) have no diagnosed hearing problems; (d) do not take cortisone for medical reasons; and (e) have a BMI < 35. The study has been approved by the Ethics Committee of the Swiss Federal Institute of Technology Zurich (Ref No. EK 2021-N-211 of 27.01.2022). The study results will be released to the participants and the public. All data will be identified with an identification number to maintain participant confidentiality. Access to the study data will be restricted to the study team, and all forms related to the study data will be kept in locked cabinets*.*

### Sample size calculation

A statistical power calculation was conducted for the restoration outcome scale as the primary outcome. A previous study, employing a similar design to the current study, was conducted by Tyrväinen et al. [14], who compared the restorative effects of exposure to a forest, a park, or an urban built environment using the restoration outcome scale. Participants sat in the respective environment for 15 min, followed by a 30-min walk. Based on the mean scores and standard deviations for the restorative outcome scale before and after the walk in the urban built environment and in the forest, reported by Tyrväinen et al. [14] (mean ROS urban built before = 4.56 (SD = 0.89), mean ROS urban built after = 4.2 (SD = 0.97), mean ROS forest before = 4.6 (SD = 0.84), mean ROS forest after = 5.16 (SD = 0.82)), the power for the current study was calculated using a simulation-based approach. For the simulation, a linear mixed effects model was used, taking group effects and participant effects into account by including a random intercept for participant groups as well as for individual participants. The required number of groups was estimated for a desired statistical power of ≥ 80%, calculating the number of simulations with a significant interaction effect of environment × time using R [100]. Power calculation showed that 19 groups with 1–6 participants per group are required to identify statistical differences between the conditions, assuming a power of ≥ 80% and an alpha level of 0.05. Thus, to accomplish an equal number of groups in urban built environments and forests, we are aiming for a minimum of 10 groups in urban built environments and 10 in forests.

### Randomization

Individuals eligible for participation can register for the study via a link to an online questionnaire using SoSci Survey [101]. Here, participants must confirm that they meet the inclusion criteria for participating in the study. Then, they will be randomly allocated to one of the study conditions (urban built with high road traffic noise, urban built with low road traffic noise, forest with high road traffic noise, forest with low road traffic noise, and forest with low traffic noise with a mindfulness intervention [the latter is not treated here]). Randomization will be conducted by the random generator within SoSci Survey, showing participants a link to an online scheduling tool [102] with free timeslots for the corresponding condition. Alternatively, participants can book a slot via e-mail or telephone. In this case, randomization is conducted by the study team staff clicking on the random generator within SoSci Survey and proposing the participant-free slots shown by the online scheduling tool in the respective condition.

### Data processing and statistical analysis

We will analyze research questions 1–5 using linear mixed-effects models. We will conduct prespecified models comparing the change from t1 to t2 in the outcome measures between participants in the different conditions. Prior to the analyses, the distributions of all measures will be examined, and data will be checked for influential outliers. The model diagnostics of linear mixed-effects models will be examined to assure that model assumptions are met. Due to the large number of outcome variables and the distinct theoretical background of the outcome variables regarding exposure to nature, the results will be analyzed and published in separate papers. One paper will focus on restoration, stress, attention, and affect. A second paper will focus on rumination.

To include road traffic noise as a predictor in the statistical model, the audio files recorded during every walk will be checked by listening to every recoding and categorizing each one-second time period in which road traffic noise is the dominant sound source. We will then calculate the *L*_Aeq_ (equivalent continuous sound level) for the cumulative time periods in which traffic noise is the dominant sound source as well as the cumulative duration in seconds in which traffic noise is dominant. Next, we will calculate the proportion of time in which road traffic noise is not the dominant sound source in relation to the total time of the recording. Then, we will compute the* L*_AE_ (sound exposure level) for the cumulative time periods in which traffic noise is dominant. Further, we will calculate the L_AE_ for the time periods when traffic noise is not dominant, using 30 dB for times when traffic noise is not dominant. This will result in a *L*_AE_ value including *L*_AE_ when traffic noise is dominant and *L*_AE_ for time periods when traffic noise is not dominant, as well as a corresponding *L*_Aeq_ value including times when traffic noise is dominant and not dominant. Noise will be included in the model as a combined predictor of *L*_AE_ including times when traffic noise is dominant and not dominant and the proportion of time, during which traffic noise is not dominant or by using the *L*_Aeq_ including times when traffic noise is dominant and not dominant. We plan to conduct a first model with vegetation and noise as categorial predictors (forest-urban built; high-low traffic noise) and a second model including noise as a continuous and vegetation as a categorial predictor.

In both models, we will specify fixed effects for conditions (vegetation and noise) and time (before and after the walk). Further, we will include an interaction term between vegetation and noise, as well as a random intercept term accounting for individual differences (one per subject). Additionally, in both models, the group constituting the experimental unit in this study will be entered as a second random intercept (one per group) to account for the hierarchical structure of the data due to the organization of participants into different groups. Since we will be conducting group walks, individuals will be subsamples of the walking groups, and the treatment will be applied to groups, not to individual participants. Therefore, the following model will be used: $outcome \sim vegetation\times noise (1 |\mathrm{ group}/\mathrm{ subject})$. If suitable, further exploratory models will be computed. For all analyses, we will set the significance level at .05 and analyses will be conducted with R [100] and R package lme4 [103].

## Discussion

This article describes the development and design of a protocol for a randomized, controlled longitudinal intervention study, comparing the effects of walking in forests and urban built environments on restoration, considering road traffic noise exposure during the walks in the respective settings.

Our planned study has several methodological strengths, including a longitudinal design, a multi-method outcome assessment including physiological measures (e.g., salivary cortisol), an attention test, and self-report data. This study extends upon previous studies comparing the effects of walking in greenspaces versus urban built environments by controlling for the potential detrimental effects of different road traffic noise levels in the environments. Since previous studies on the effects of noise on stress have often been based on laboratory [44, 51, 55–57] or cross-sectional data [41, 43, 53, 54], this study contributes to emerging research in providing data from a longitudinal field study. Unlike previous studies, which only assessed noise at one representative point on each route in each environment [40], noise will be measured continuously throughout every walk to assess the effect of the effective noise in the actual moment of the walk.

Further, rather than focusing on restoration from a stressed or depleted state, we will explore the instorative effects of walking in different environments with participants who have not undergone a prior stress intervention [104, 105]. Many previous studies have examined restoration after stressing participants [68, 74, 106, 107], which makes it easier to find greater restoration effects because of a higher baseline level of stress and attentional fatigue [89, 104]. However, the aim of this study is to examine the potential of walking in different environments for the psychophysiological wellbeing of individuals with an ecologically valid amount of stress and the need for restoration in their everyday life, without an artificial induction of stress or fatigue. This is because we focus on exposure to greenspaces as a low-threshold approach in the general public, not merely focusing on reducing stress and restoring depleted attentional resources but also on fostering wellbeing.

This study also has some methodological limitations. First, while we expect the settings in noisy conditions to display higher mean noise values compared to relatively quiet conditions, mean noise levels in urban built settings are expected to be systematically louder than in forest settings. When beginning the process of selecting the test sites, we aimed to apply the same noise thresholds in terms of dBA for loud and quiet urban built and forest settings for the GIS analysis, as we wanted to compare these. During the selection process of the test settings, however, it became clear that defining loud and quiet depends on the context of the site, with mean noise levels in urban built environments being, on average, higher than in forest settings (see Table 2). This systematic difference between urban built environments and forests, however, if not explicitly accounted for in the statistical model, might result in a potential systematic confounding of noise level and setting category (urban built or forest). We therefore plan to account for this by including noise as a continuous predictor in the second model, as discussed above. However, this must be considered in the data analysis and interpretation of the study results.

Second, because noise features high situational variance, finding situations with widely constant noise conditions was a major challenge. Thus, the noise variance within each setting will have to be considered and discussed along with the final data analysis. The same also applies to seasonal differences in the vegetation and thus in VHM and NDVI.

Third, it is important to note that without further information on the type of sound source, dB values possess only limited potential to predict noise annoyance and consequently stress. The assessed noise levels during the walks will partly reflect sound from individual motorized traffic but also other sound sources, such as sounds from people, animals, wind, and moving leaves from trees. The research team, with expertise in both environmental and health psychology as well as acoustics research, carefully discussed these limitations during the study design process; however, there is no viable way to fully avoid them in a field study. Therefore, we plan to account for this by extracting the percentage of time, during which road traffic noise is the dominant sound source in the sound measurements of every walk, as well as by including the *L*_Aeq_ during the time when road traffic noise is the dominating sound source as predictors for the statistical model. Fourth, especially when measuring sound during the walks in the forest settings, the sound of the experimenter walking partly masks other, quieter, or more distant sounds. Thus, the *L*_Aeq_ from such sounds (e.g., natural sounds) during the walks cannot be extracted from the recordings. However, because traffic noise is usually louder than the walking sound, this will only be a minor source of uncertainty regarding the recorded road traffic *L*_Aeq_ during the walks.

Finally, for individuals working full time, who could be hypothesized to be the ones with the highest stress levels, it might be difficult to find a convenient, appropriate, or suitable time slot, since walking sessions only take place in the afternoon.

The results of this study will inform us about the restorative effects of exposure to different types of landscape environments and to different road traffic noise levels at these sites. Through the methodology of this study, this research not only adds to the body of literature on the topic, but also provides an improved foundation for practical applications. The study will add information about the benefits of walking and nature-based interventions as well as their potential to reduce stress and promote wellbeing, thus facilitating a better understanding of low-threshold interventions to prevent stress and foster wellbeing. In addition, the results will have the potential to inform noise legislation and the implementation of spatial planning acts.

### Supplementary Information

**Additional file 1. **Locations of the starting points of the walking routes in the test settings.

**Additional file 2. **Data on *L*_Aeq_ from noise recordings of walking routes.

**Additional file 3. **Data on the evaluation of acoustic impression of the test settings.

**Additional file 4. **Participant information sheet.

**Additional file 5. **Participant consent form.

**Additional file 6. **Completed SPIRIT checklist.

**Additional file 7. **GPS tracks of walking routes.

## Data Availability

The data from the setting selection process is publicly available on the Open Science Framework under https://osf.io/2bfgv/. The final dataset of the study will be made publicly available in the results publications.
