# Supplementary material for: Psychophysiological effects of walking in forests and urban built environments with disparate road traffic noise exposure: study protocol of a randomized controlled trial
Source: BMC Psychol. 2024 May 6;12:250. doi: 10.1186/s40359-024-01720-x (PMC11073983; doi:10.1186/s40359-024-01720-x)
Supplement: Supplementary file 1 — Additional file 1. Locations of the starting points of the walking routes in the test settings. [file 40359_2024_1720_MOESM1_ESM.pdf]

Locations of the starting points of the walking routes in the test settings:

1. Wydlerweg 6, 8047 Zürich. https://goo.gl/maps/QKtxnumGwZNTHfEq5, 47.375919, 8.485517. 47°22'33.3"N 8°29'07.9"E.
2. Albisriederstrasse 391, 8047 Zürich. https://goo.gl/maps/Jo644y8oRkWGBuH89, 47374555, 8.483707. 47°22'28.4"N 8°29'01.4"E.
3. Schweighofstrasse 292, 8055 Zürich. https://goo.gl/maps/UhKgRjKNUrMZuYNZ7, 47.362668, 8.504677. 47°21'45.6"N 8°30'16.8"E.
4. Rieterplatz, 8002 Zürich. https://goo.gl/maps/cM39UG58FyaHS1yFA, 47.356475, 8.526407, 47°21'23.3"N 8°31'35.1"E.
5. Saumackerstr. 83, 8048 Zürich. https://goo.gl/maps/drx1Ysfe5FdRDzu5A, 47.384338, 8.488079, 47°23'03.6"N 8°29'17.1"E.
6. Langgrütstrasse 137, 8047 Zürich. https://goo.gl/maps/ayzuGXJ362RjTS257 47.373670, 8.492139, 47°22'25.2"N 8°29'31.7"E.
7. Krähbühlweg, 8044 Zürich. https://goo.gl/maps/kAWHjiYRUBrxcSMq8 47.378480, 8.572203, 47°22'42.5"N 8°34'19.9"E.
8. Bruderholzweg, 8053 Zürich. https://goo.gl/maps/jukedSoKBW4w1epv8 47.366104, 8.598972, 47°21'58.0"N 8°35'56.3"E.
9. Alte Regensdorferstr., 8049 Zürich. https://goo.gl/maps/vcAWNwwLCnVao1FR8, 47.416268, 8.484467, 47°24'58.6"N 8°29'04.1"E.
10. Hermann-Trüb-Weg, 8044 Dübendorf. https://goo.gl/maps/LBoeMaQCMET86nTT6, 47.379004, 8.597841, 47°22'44.4"N 8°35'52.2"E.
11. Forsthausweg, 8044 Zürich. https://goo.gl/maps/2HRYtZVJdpf9LMRN9, 47.380767, 8.589668, 47°22'50.8"N 8°35'22.8"E.
12. Hungerbergstr., 8049 Zürich. https://goo.gl/maps/q49w7woo6oxxx8yw6, 47.416988, 8.495863, 47°25'01.2"N 8°29'45.1"E.
