# Supplementary material for: Psychophysiological effects of walking in forests and urban built environments with disparate road traffic noise exposure: study protocol of a randomized controlled trial
Source: BMC Psychol. 2024 May 6;12:250. doi: 10.1186/s40359-024-01720-x (PMC11073983; doi:10.1186/s40359-024-01720-x)
Supplement: Supplementary file 4 — Additional file 4. Participant information sheet. [file 40359_2024_1720_MOESM4_ESM.pdf]

Eidg. Forschungsanstalt WSL
Institut fédéral de recherches WSL
Istituto federale di ricerca WSL
Swiss Federal Research Institute WSL

Allgemeine Teilnehmendeninformation „Studie zu förderlichen und hinderlichen Faktoren für die Erholung bei halbstündigen Spaziergängen“

1. Was ist das Ziel der Studie?

Verschiedene Studien deuten darauf hin, dass Spazierengehen in Abhängigkeit von der Umgebung, in der Menschen spazieren gehen sowie je nach Haltung, mit der Menschen der Umgebung begegnen unterschiedlich stark zu Erholung beitragen kann. In diesem Sinne untersucht die Studie unter welchen Bedingungen Spaziergänge eine erholsame Wirkung haben. Die Studie ist Teil des Forschungsprojekts RESTORE, das von der Eidg. Forschungsanstalt für Wald, Schnee und Landschaft (WSL) koordiniert und durch den Schweizerischen Nationalfonds (SNF) finanziert wird.
Das *erste Untersuchungsziel* der Studie besteht darin zu erforschen, welchen Einfluss Verkehrslärm und Vegetation für die Erholung beim Spazierengehen haben. Die Ergebnisse sollen Aufschluss über das Erholungspotenzial unterschiedlicher Umgebungen geben. Im Rahmen *des zweiten Untersuchungsziels* wird untersucht, ob neben Umgebungsfaktoren auch die innere Haltung, mit der Menschen der Umgebung beim Spazierengehen begegnen, einen Einfluss auf die Erholung bei Spaziergängen hat.

2. Wie sieht der Ablauf der Studie aus?

Die Studie umfasst einen halbstündigen, geleiteten Spaziergang in einer kleinen Gruppe (2-6 Personen) auf einer von uns vorgegebenen Strecke.
Vor Ort lesen Sie zunächst aufmerksam diese Teilnehmerinformation und werden dann aufgefordert Ihr Einverständnis zur Studienteilnahme zu geben. Anhand von Speichelproben messen wir vor und nach dem Spaziergang das Hormon Cortisol, welches das Ausmass an Stress anzeigt. Zudem wird während des Spaziergangs die körperliche Anspannung mit Hilfe eines Geräts zur Erfassung des Hautleitwerts gemessen. Dafür werden Elektroden zur Erfassung des Hautleitwerts an einer Ihrer Hände angeklebt und während des 30-minütigen Spaziergangs vor Ort getragen. Nach dem Spaziergang werden diese schmerzfrei wieder entfernt. Vor und nach dem Spaziergang bitten wir Sie ausserdem eine Aufgabe zu bearbeiten, die Aufmerksamkeit erfordert und fragen sie in einem Fragebogen nach Ihrem Befinden, mentalen Zuständen, Persönlichkeitseigenschaften, momentaner Aufmerksamkeit, Formen des Nachdenkens, demographischen Informationen und ihrem Eindruck von der Umgebung. Sie erhalten von uns eine persönliche ID-Nummer, die Sie in alle Fragebögen eintragen. Während des Spaziergangs folgen Sie einfach den Anweisungen der Studienleitung, sie weist Ihnen auch den Weg.
Im Anschluss an den Spaziergang vor Ort bitten wir Sie, in den nächsten 10 Tagen noch drei Mal für eine halbe Stunde in einer Umgebung mit bestimmten Eigenschaften spazieren zu gehen. Sie bekommen von der Studienleitung vor Ort genauere Informationen hierzu. Nach 10 Tagen senden wir Ihnen per E-Mail eine Einladung mit einem Link zu einem dritten Fragebogen zu, in den es erneut um Ihre Befindlichkeit geht. Wir führen die Studie im Einklang mit den Schweizer Gesetzen durch. Die Ethikkommission der ETH hat die Studie geprüft und bewilligt.

3. Welchen Nutzen hat die Studienteilnahme für Sie?

Die Eidgenössische Forschungsanstalt für Wald, Schnee und Landschaft WSL erarbeitet Grundlagen für eine bedürfnisgerechte, nachhaltige Landschaftsentwicklung und Raumplanung. Durch Ihre Teilnahme ermöglichen Sie uns die Untersuchung von förderlichen und hinderlichen Faktoren wie beispielsweise Verkehrslärm und Vegetation für die Erholung bei Spaziergängen. Mit den Ergebnissen hoffen wir einen Beitrag leisten zu können, dass die Landschaft so gestaltet wird, dass auch wir Menschen uns darin wohl fühlen und erholen können. Dank Ihrer Studienteilnahme können die Ergebnisse der Allgemeinbevölkerung zugutekommen – vor allem dann, wenn über städtische Natur und die Zukunft städtischer Flächen für Erholungszwecke verhandelt wird. Mit der Studie sind keine bekannten Risiken oder Belastungen verbunden.

4. Rechte und Pflichten der Teilnehmenden

Sie nehmen an der Studie nur teil, wenn *Sie* es wollen. Sie können Ihren Entscheid jederzeit und ohne Begründung zurücknehmen. Sie dürfen jederzeit Fragen zur Studie stellen; unsere Kontaktdaten finden Sie am Ende dieser Studieninformation.

5. Vertraulichkeit der Daten und Proben

Für diese Studie erfassen wir Daten zu Ihrer Befindlichkeit, Persönlichkeit und weiteren Eigenschaften (siehe oben). Zudem untersuchen wir Ihren Cortisol- und Hautleitwert. Diese Daten und Proben werden anonymisiert. Das bedeutet, dass wir alle Angaben, die Sie identifizieren könnten durch einen Code ersetzen, der keine Rückschlüsse auf Ihre Person ermöglicht. Wir werden Ihnen deshalb Ihre persönlichen Ergebnisse nicht mitteilen können. Ihren Namen werden wir nicht veröffentlichen, weder in einem Bericht, einer Publikation, noch in sonst einer Weise gedruckt oder im Internet. Alle Personen, die mit der Studie in irgendeiner Weise zu tun haben, verpflichten sich zu absoluter Vertraulichkeit. Am Ende der Studie vernichten wir die Proben, die Daten bewahren wir nach den gesetzlichen Vorgaben auf. Die Teilnahme an der Studie ist freiwillig.

6. Deckung von Schäden

Allfällige Gesundheitsschäden, die in direktem Zusammenhang mit der Studie entstehen und auf Verschulden der WSL zurückzuführen sind, sind durch eine Betriebshaftpflichtversicherung gedeckt (Basler Versicherungen, Police Nr. 30 5 007 450 6). Der Abschluss einer Unfall-/Krankenversicherung liegt in der Verantwortung des Probanden. Wenn Sie einen Schaden erlitten haben, wenden Sie sich bitte an die Leiterin der Studie.

7. Kontaktpersonen

Bei Unklarheiten oder Bedenken, die während oder nach der Studie auftreten, können Sie sich jederzeit an eine der folgenden Kontaktpersonen wenden:

Studiendurchführung: Julia Schaupp (julia.schaupp@wsl.ch, Tel.: 044 739 24 84)
Leiterin der Studie: Dr. Nicole Bauer (nicole.bauer@wsl.ch, Tel: 044 739 24 58)
