# Supplementary material for: Psychophysiological effects of walking in forests and urban built environments with disparate road traffic noise exposure: study protocol of a randomized controlled trial
Source: BMC Psychol. 2024 May 6;12:250. doi: 10.1186/s40359-024-01720-x (PMC11073983; doi:10.1186/s40359-024-01720-x)
Supplement: Supplementary file 5 — Additional file 5. Participant consent form. [file 40359_2024_1720_MOESM5_ESM.pdf]

Eidg. Forschungsanstalt WSL
Institut fédéral de recherches WSL
Istituto federale di ricerca WSL
Swiss Federal Research Institute WSL

Schriftliche Einwilligungserklärung zur Teilnahme an der Studie zu förderlichen und hinderlichen Faktoren für die Erholung bei halbstündigen Spaziergängen

- Bitte lesen Sie diese Seite sorgfältig durch.
- Bitte fragen Sie, wenn Sie etwas nicht verstehen. Wir geben gerne Auskunft.

**Titel der Studie**	„Hinderliche und förderliche Faktoren für die Erholung bei Spaziergängen in unterschiedlichen Umgebungen“
**Verantwortliche Institution**	Eidg. Forschungsanstalt für Wald, Schnee und Landschaft WSL, Zürcherstrasse 111, 8903 Birmensdorf
**Ort der Durchführung**	Raum Zürich
**Leiterin der Studie**	Dr. Nicole Bauer

- Ich wurde über den Zweck und Ablauf der Studie sowie über eventuelle Risiken informiert.
- Ich nehme an dieser Studie freiwillig teil. Ich kann jederzeit und ohne Angabe von Gründen meine Zustimmung zur Teilnahme widerrufen, ohne dass mir deswegen Nachteile entstehen.
- Ich habe das Dokument mit der Teilnehmendeninformation gelesen. Meine Fragen im Zusammenhang mit der Teilnahme an dieser Studie sind mir zufriedenstellend beantwortet worden. Ich kann die schriftliche Studieninformation behalten, ebenso wie eine Kopie dieser Einwilligungserklärung.
- Ich akzeptiere den Inhalt der zur oben genannten Studie abgegebenen schriftlichen Studieninformation.
- Ich hatte genügend Zeit, meine Entscheidung zu treffen.
- Ich bin darüber informiert, dass eine Versicherung Schäden deckt, die mir durch Teilnahme an dieser Studie entstanden sind.
- Mit meiner Unterschrift bestätige ich, dass ich die im Informationsblatt genannten Voraussetzungen für die Studienteilnahme erfülle.
- Ich bin darüber informiert, dass eine Versicherung Schäden deckt, die in direktem Zusammenhang mit der Studie entstehen und auf Verschulden der WSL zurückzuführen sind.
- Ich weiss, dass meine persönlichen Daten und Proben nur in anonymisierter Form für dieses Forschungsprojekt genutzt werden. Ich bin einverstanden, dass die zuständigen Fachleute des Auftraggebers der Studie, der Behörden und der für diese Studie zuständigen Ethikkommission zu Prüf- und Kontrollzwecken in meine

Originaldaten Einsicht nehmen dürfen, jedoch unter strikter Einhaltung der Vertraulichkeit.

**Teilnehmerin/Teilnehmer**	Vollständiger Name:<br>Geburtsdatum:
Ort, Datum	Unterschrift Studienteilnehmerin/Studienteilnehmer
