# Supplementary material for: Psychophysiological effects of walking in forests and urban built environments with disparate road traffic noise exposure: study protocol of a randomized controlled trial
Source: BMC Psychol. 2024 May 6;12:250. doi: 10.1186/s40359-024-01720-x (PMC11073983; doi:10.1186/s40359-024-01720-x)
Supplement: Supplementary file 6 — Additional file 6. Completed SPIRIT checklist. [file 40359_2024_1720_MOESM6_ESM.pdf]

Reporting checklist for protocol of a clinical trial.

Based on the SPIRIT guidelines.

Instructions to authors

Complete this checklist by entering the page numbers from your manuscript where readers will find each of the items listed below.

Your article may not currently address all the items on the checklist. Please modify your text to include the missing information. If you are certain that an item does not apply, please write "n/a" and provide a short explanation.

Upload your completed checklist as an extra file when you submit to a journal.

In your methods section, say that you used the SPIRITreporting guidelines, and cite them as:

Chan A-W, Tetzlaff JM, Gøtzsche PC, Altman DG, Mann H, Berlin J, Dickersin K, Hróbjartsson A, Schulz KF, Parulekar WR, Krleža-Jerić K, Laupacis A, Moher D. SPIRIT 2013 Explanation and Elaboration: Guidance for protocols of clinical trials. BMJ. 2013;346:e7586

		Reporting Item	Page Number
**Administrative information**			
Title	#1	Descriptive title identifying the study design, population, interventions, and, if applicable, trial acronym	title page
Trial registration	#2a	Trial identifier and registry name. If not yet registered, name of intended registry	2
Trial registration: data set	#2b	All items from the World Health Organization Trial Registration Data Set	2
Protocol version	#3	Date and version identifier	n/a, until now, no amendments were made

Funding	#4	Sources and types of financial, material, and other support	35
Roles and responsibilities: contributorship	#5a	Names, affiliations, and roles of protocol contributors	35, title page
Roles and responsibilities: sponsor contact information	#5b	Name and contact information for the trial sponsor	35
Roles and responsibilities: sponsor and funder	#5c	Role of study sponsor and funders, if any, in study design; collection, management, analysis, and interpretation of data; writing of the report; and the decision to submit the report for publication, including whether they will have ultimate authority over any of these activities	35
Roles and responsibilities: committees	#5d	Composition, roles, and responsibilities of the coordinating centre, steering committee, endpoint adjudication committee, data management team, and other individuals or groups overseeing the trial, if applicable (see Item 21a for data monitoring committee)	35
**Introduction**			
Background and rationale	#6a	Description of research question and justification for undertaking the trial, including summary of relevant studies (published and unpublished) examining benefits and harms for each intervention	7-9, 2-7

Background and rationale: choice of comparators	#6b	Explanation for choice of comparators	3-6
Objectives	#7	Specific objectives or hypotheses	5-7,7-9
Trial design	#8	Description of trial design including type of trial (eg, parallel group, crossover, factorial, single group), allocation ratio, and framework (eg, superiority, equivalence, non-inferiority, exploratory)	7
**Methods: Participants, interventions, and outcomes**			
Study setting	#9	Description of study settings (eg, community clinic, academic hospital) and list of countries where data will be collected. Reference to where list of study sites can be obtained	21-26
Eligibility criteria	#10	Inclusion and exclusion criteria for participants. If applicable, eligibility criteria for study centres and individuals who will perform the interventions (eg, surgeons, psychotherapists)	27
Interventions: description	#11a	Interventions for each group with sufficient detail to allow replication, including how and when they will be administered	9-12
Interventions: modifications	#11b	Criteria for discontinuing or modifying allocated interventions for a given trial participant (eg, drug dose change in response to harms, participant	27

		request, or improving / worsening disease)	
Interventions: adherance	#11c	Strategies to improve adherence to intervention protocols, and any procedures for monitoring adherence (eg, drug tablet return; laboratory tests)	27
Interventions: concomitant care	#11d	Relevant concomitant care and interventions that are permitted or prohibited during the trial	n/a, no concomitant care
Outcomes	#12	Primary, secondary, and other outcomes, including the specific measurement variable (eg, systolic blood pressure), analysis metric (eg, change from baseline, final value, time to event), method of aggregation (eg, median, proportion), and time point for each outcome. Explanation of the clinical relevance of chosen efficacy and harm outcomes is strongly recommended	12-18
Participant timeline	#13	Time schedule of enrolment, interventions (including any run-ins and washouts), assessments, and visits for participants. A schematic diagram is highly recommended (see Figure)	12
Sample size	#14	Estimated number of participants needed to achieve study objectives and how it was determined, including clinical and statistical assumptions supporting any sample size calculations	28

Recruitment	#15	Strategies for achieving adequate participant enrolment to reach target sample size		27
**Methods: Assignment of interventions (for controlled trials)**				
Allocation: sequence generation	#16a	Method of generating the allocation sequence (eg, computer-generated random numbers), and list of any factors for stratification. To reduce predictability of a random sequence, details of any planned restriction (eg, blocking) should be provided in a separate document that is unavailable to those who enrol participants or assign interventions		29
Allocation concealment mechanism	#16b	Mechanism of implementing the allocation sequence (eg, central telephone; sequentially numbered, opaque, sealed envelopes), describing any steps to conceal the sequence until interventions are assigned		29
Allocation: implementation	#16c	Who will generate the allocation sequence, who will enrol participants, and who will assign participants to interventions		29
Blinding (masking)	#17a	Who will be blinded after assignment to interventions (eg, trial participants, care providers, outcome assessors, data analysts), and how	n/a: blinding of participants is not possible because of obvious differences between the interventions, nevertheless participants are not told, which condition they were randomized to	

Blinding (masking): emergency unblinding	#17b	If blinded, circumstances under which unblinding is permissible, and procedure for revealing a participant's allocated intervention during the trial	n/a due to the nature of the study, neither participants nor the tinvestigators can be completely blinded.
**Methods: Data collection, management, and analysis**			
Data collection plan	#18a	Plans for assessment and collection of outcome, baseline, and other trial data, including any related processes to promote data quality (eg, duplicate measurements, training of assessors) and a description of study instruments (eg, questionnaires, laboratory tests) along with their reliability and validity, if known. Reference to where data collection forms can be found, if not in the protocol	12-18
Data collection plan: retention	#18b	Plans to promote participant retention and complete follow-up, including list of any outcome data to be collected for participants who discontinue or deviate from intervention protocols	27 and questionnaire 3 in supplementary information
Data management	#19	Plans for data entry, coding, security, and storage, including any related processes to promote data quality (eg, double data entry; range checks for data values). Reference to where details of data management procedures can be found, if not in the protocol	27-28
Statistics: outcomes	#20a	Statistical methods for analysing primary and secondary outcomes. Reference to where other details of	29-30

		the statistical analysis plan can be found, if not in the protocol	
Statistics: additional analyses	#20b	Methods for any additional analyses (eg, subgroup and adjusted analyses)	29-30
Statistics: analysis population and missing data	#20c	Definition of analysis population relating to protocol non-adherence (eg, as randomised analysis), and any statistical methods to handle missing data (eg, multiple imputation)	29-30
**Methods: Monitoring**			
Data monitoring: formal committee	#21a	Composition of data monitoring committee (DMC); summary of its role and reporting structure; statement of whether it is independent from the sponsor and competing interests; and reference to where further details about its charter can be found, if not in the protocol. Alternatively, an explanation of why a DMC is not needed	n/a: no data monitoring committee, due to known minimal risk
Data monitoring: interim analysis	#21b	Description of any interim analyses and stopping guidelines, including who will have access to these interim results and make the final decision to terminate the trial	n/a: due to known minimal risk
Harms	#22	Plans for collecting, assessing, reporting, and managing solicited and spontaneously reported adverse events and other unintended effects of trial interventions or trial conduct	n/a: known minimal risk for participants, but we ask an open question in the questionnaire after the walk, where participants can put in what was important for them, if they want to add anything not asked in the questionnaire.

Auditing	#23	Frequency and procedures for auditing trial conduct, if any, and whether the process will be independent from investigators and the sponsor	n/a: no auditing independent from investigators
**Ethics and dissemination**			
Research ethics approval	#24	Plans for seeking research ethics committee / institutional review board (REC / IRB) approval	27
Protocol amendments	#25	Plans for communicating important protocol modifications (eg, changes to eligibility criteria, outcomes, analyses) to relevant parties (eg, investigators, REC / IRBs, trial participants, trial registries, journals, regulators)	n/a: no ammendments yet
Consent or assent	#26a	Who will obtain informed consent or assent from potential trial participants or authorised surrogates, and how (see Item 32)	9, 35-36, supplementary file
Consent or assent: ancillary studies	#26b	Additional consent provisions for collection and use of participant data and biological specimens in ancillary studies, if applicable	n/a: supplementary file
Confidentiality	#27	How personal information about potential and enrolled participants will be collected, shared, and maintained in order to protect confidentiality before, during, and after the trial	27-28
Declaration of interests	#28	Financial and other competing interests for principal investigators for the overall trial and each study site	35

Data access	#29	Statement of who will have access to the final trial dataset, and disclosure of contractual agreements that limit such access for investigators	35
Ancillary and post trial care	#30	Provisions, if any, for ancillary and post-trial care, and for compensation to those who suffer harm from trial participation	n/a: no ancillary care due to known minimal risk
Dissemination policy: trial results	#31a	Plans for investigators and sponsor to communicate trial results to participants, healthcare professionals, the public, and other relevant groups (eg, via publication, reporting in results databases, or other data sharing arrangements), including any publication restrictions	27
Dissemination policy: authorship	#31b	Authorship eligibility guidelines and any intended use of professional writers	34-35, no use of professional writers; substantive contributions to the design, conduct, interpretation, and reporting of the study will be recognised through the granting of authorship on the results paper
Dissemination policy: reproducible research	#31c	Plans, if any, for granting public access to the full protocol, participant-level dataset, and statistical code	35
**Appendices**			
Informed consent materials	#32	Model consent form and other related documentation given to participants and authorised surrogates	n/a: supplementary file
Biological specimens	#33	Plans for collection, laboratory evaluation, and storage of biological	12, no storage for future or ancillary studies

specimens for genetic or molecular analysis in the current trial and for future use in ancillary studies, if applicable

Notes:

- 1: n/a: see title page
- 3: n/a, until now, no amendments were made
- 5a: 35, title page
- 11d: n/a, no concomitant care
- 17a: n/a: blinding of participants is not possible because of obvious differences between the interventions, nevertheless participants are not told, which condition they were randomized to
- 17b: n/a due to the nature of the study, neither participants nor the tinvestigators can be completely blinded.
- 18b: 27 und questionnaire 3 in supplementary information
- 21a: n/a: no data monitoring committee, due to known minimal risk
- 21b: n/a: due to known minimal risk
- 22: n/a: known minimal risk for participants, but we ask an open question in the questionnaire after the walk, where participants can put in what was important for them, if they want to add anything not asked in the questionnaire.
- 23: n/a: no auditing independent from investigators
- 25: n/a: no ammendments yet
- 26a: 9, 35-36, supplementary file
- 26b: n/a: supplementary file
- 30: n/a: no ancillary care due to known minimal risk
- 31b: 34-35, no use of professional writers; substantive contributions to the design, conduct, interpretation, and reporting of the study will be recognised through the granting of authorship on the results paper
- 32: n/a: supplementary file

- 33: 12, no storage for future or ancillary studies The SPIRIT Explanation and Elaboration paper is distributed under the terms of the Creative Commons Attribution License CC-BY-NC. This checklist was completed on 27. November 2023 using https://www.goodreports.org/, a tool made by the EQUATOR Network in collaboration with Penelope.ai
